# Ultrafast and Slow Cholinergic Transmission. Different Involvement of Acetylcholinesterase Molecular Forms †

**DOI:** 10.3390/molecules22081300

**Published:** 2017-08-04

**Authors:** Yves Dunant, Victor Gisiger

**Affiliations:** 1 Département des Neurosciences Fondamentales, Faculté de Médecine, Université de Genève, CH-1211-Genève 4, Switzerland; 2 Département de Pathologie et Biologie Cellulaire, Faculté de Médecine, Université de Montréal, Montréal, QC H3C 3J7, Canada; victor.gisiger@umontreal.ca

**Keywords:** acetylcholine, acetylcholinesterase, neuromuscular junction, electric organ synaptic transmission, cholinergic synapses, non-neuronal acetylcholine

## Abstract

Acetylcholine (ACh), an ubiquitous mediator substance broadly expressed in nature, acts as neurotransmitter in cholinergic synapses, generating specific communications with different time-courses. (1) *Ultrafast transmission*. Vertebrate neuromuscular junctions (NMJs) and nerve-electroplaque junctions (NEJs) are the fastest cholinergic synapses; able to transmit brief impulses (1–4 ms) at high frequencies. The collagen-tailed A12 acetylcholinesterase is concentrated in the synaptic cleft of NMJs and NEJs, were it curtails the postsynaptic response by ultrafast ACh hydrolysis. Here, additional processes contribute to make transmission so rapid. (2) *Rapid transmission*. At peripheral and central cholinergic neuro-neuronal synapses, transmission involves an initial, relatively rapid (10–50 ms) nicotinic response, followed by various muscarinic or nicotinic effects. Acetylcholinesterase (AChE) being not concentrated within these synapses, it does not curtail the initial rapid response. In contrast, the late responses are controlled by a globular form of AChE (mainly G4-AChE), which is membrane-bound and/or secreted. (3) *Slow ACh signalling.* In non-neuronal systems, in muscarinic domains, and in most regions of the central nervous system (CNS), many ACh-releasing structures (cells, axon terminals, varicosities, boutons) do not form true synaptic contacts, most muscarinic and also part of nicotinic receptors are extra-synaptic, often situated relatively far from ACh releasing spots. A12-AChE being virtually absent in CNS, G4-AChE is the most abundant form, whose function appears to modulate the “volume” transmission, keeping ACh concentration within limits in time and space.

## 1. Introduction

Acetylcholine (ACh) has been recognised as a neurotransmitter, initially in organs innervated by the parasympathetic nervous system [1], then in neuromuscular junctions and in neuro-neuronal synapses of autonomic and central nervous systems [2,3]. However, it has long be known that significant amounts of ACh are present in non-neuronal tissues, such as, the corneal epithelium, the human placenta, and even in plants like the nettles (*Urtica urens*). Actually, more recent research revealed that cholinergic signalling was established from the very beginning of life; that is, in primitive unicellular and multicellular organisms, in bacteria, algae, plants and fungi [4,5]. It is therefore not surprising that in vertebrates ACh is synthesised and stored in a variety of non-neuronal cells, from which the mediator is released in a paracrine manner. We are just beginning to understand the physiological significance and the molecular mechanisms of this type of non-neuronal cholinergic signalling, notably in lymphocytes [6], vascular endothelial cells [7], rat kidney cells [8], human pancreatic alpha-cells [9], and in reproductive apparatus of bees and humans [7], among other systems.

The role of ACh as neurotransmitter in neuro-neuronal synapses of the autonomic (ANS) and the central nervous systems (CNS) has been better documented, but recent investigations revealed that transmission in these places is much more subtle and complex than initially believed. A stimulus applied to the afferent nerve fibres evokes an initial, relatively rapid, postsynaptic response (Excitatory Post-Synaptic Potential, EPSP), which is followed by a host of slower responses involving various types of muscarinic, nicotinic and other receptors. In these synapses, acetylcholinesterase (AChE) is not concentrated in the narrow cleft separating pre- and postsynaptic membranes.

In neuromuscular (NMJ) and nerve-electroplaque (NEJ) junctions the picture is quite different. Transmission of an individual impulse is 10–50 times faster than in cholinergic neuro-neuronal synapses. A number of specialized features work together to shorten the flash-like impulses, enabling these junctions to fire at a very high frequency.

In this diversity of cholinergic mechanisms, time is a crucial parameter. Cholinergic mediation can be extremely rapid (a few milliseconds) or very slow (several minutes), a range of time courses encompassing more than five orders of magnitude. Moreover, distinct processes functioning with different time courses can cooperate at the same junctional spot. A point is important to recall here. Since fast transmission must rely on rapid physicochemical reactions, these must be characterised by a low-affinity. Indeed, the velocity of a chemical reaction is inversely related to its affinity. In other words: time is gained at the expense of sensitivity, as elegantly formulated by Bernard Katz [10].

The purpose of the present paper is to describe some cytological and molecular features which make a cholinergic cell to transmit messages with either a fast or a slow time course. Peculiar attention will be focused on the role played by some molecular forms of AChE in these processes. The present knowledge on AChE molecular forms has been extensively reviewed several times and the reader will find further descriptions in the present volume. Here we will limit ourselves to focus our attention on the two groups of physiologically active AChE molecular forms, which fulfil specific functions in the different kinetics of cholinergic signalling.

First, asymmetric collagen-tailed forms (ColQ) of AChE, mainly A12-AChE, are confined to the basal lamina of the skeletal muscle NMJ [11], and *Torpedo* NEJ [12,13]. In turn, globular forms of AChE, mainly G4-AChE, predominate in the peri-junctional region outside of the NMJ of skeletal muscles [14,15]. G4-AChE is also present in cholinergic and non-cholinergic neurons and glial cells (see review in [16]). These G4 molecules are predominantly amphiphilic as a result of their association with a proline-rich membrane anchor (PRiMA) [11,17]. However, neurons and skeletal muscles both secrete large amounts of non-amphiphilic G4 molecules suggesting a specific physiological role for them [16,18,19,20].

## 2. Ultra-Fast Cholinergic Transmission

Vertebrate neuromuscular junctions are giant synapses. The contact area between axon endings and muscle cell stretches over several hundreds of µm^2^. NMJs are structured with regular differentiations, called “active zones” and characterised by a double row of synaptic vesicles [21]. Transmission is quantal in NMJs [22] with the release of approximately one quantum par active zone [23]. Neuromuscular transmission of a single impulse takes no more than 2–4 ms, even in poikilothermic animals. To command a sustained muscle contraction, NMJs have to fire at frequencies up to 100 Hz. This means that the interval between successive impulses is of the order of 10 ms. Obviously, each of the processes directly involved in rapid transmission of individual impulses must be very fast, thus relying on low-affinity reactions [10].

### 2.1. Two Different Molecular Forms of Acetylcholinesterase Are Associated with Muscle Function

A specific form of acetylcholinesterase, localised in the synaptic cleft, abbreviates neurotransmission in neuromuscular and nerve-electroplaque junctions. It has long been known that AChE is highly concentrated in the synaptic cleft of NMJs [24,25]. This junctional AChE is an asymmetric collagen-tailed form, mainly A12**-**AChE, which is anchored to the basal lamina occupying the synaptic space and following the post-junctional folds [17,26,27].

Three features confer to A12-AChE its major role in the ultra-fast cholinergic transmission: (a) its location in the centre of the NMJ junction, where it is positioned between the active zones of the motor nerve ending and the nicotinic receptors bound to the postsynaptic membrane boarding the primary cleft [15]; (b) its substrate inhibition for ACh concentrations exceeding 1 mM [28]; (c) its very high rate of ACh hydrolysis, beyond 10^3^ ACh molecules/s [26,29]. Accordingly, ACh molecules released from the active zones at almost molar concentrations [30,31] pass through the basal lamina while the anchored asymmetric AChE is temporarily inactivated by substrate inhibition [32]. ACh molecules reach the low affinity nicotinic receptors and ultimately, they are immediately destroyed when leaving them, since at that time the local ACh concentration has fallen and AChE has regained its full hydrolytic power, thus cutting short the impulse within a few ms. Consequently, reversible and irreversible cholinesterase inhibitors greatly lengthen the endplate potential (EPP) decay phase in vertebrate NMJs [33,34,35].

In addition to the asymmetric collagen-tailed forms, muscles also contain the tetrameric globular form G4 which fulfils a distinct functional role. G4-AChE is located in peri-junctional regions [14,15], as both a membrane-bound enzyme anchored by PRiMA [36], and a secreted, soluble enzyme [37]. The abundance of G4-AChE varies according to the muscle twitch properties (it is high or low, in fast and slow muscles, respectively). Moreover, the amount of G4 increases in proportion to the level of fast muscle activity, without any significant changes in the asymmetric forms (A12-AChE) [38]. This fact strengthens the conclusion that, whereas the asymmetric forms implement their functional role at the level of each individual fiber, the G4 pool, in turn, fulfils a specific global need of the entire muscle, or of an anatomical subunit if the muscle is subdivided into heads [14,39]. Indeed, the G4-rich environment that fast muscles provide at the motor zone is collectively contributed by neighbouring fibers. This strengthens the view that the functional role of the globular G4 pool is fulfilled by the part of it that is placed outside the muscle fibers, as both membrane-bound and secreted molecules [14,40].

From a functional point of view, it appears that the globular G4-AChE—which does not abbreviate the time course of individual impulses—controls the ambient concentration of ACh, created by its diffusion out of the junctions upon repetitive stimulation; G4 therefore is expected to protect nicotinic receptors from desensitization [14,16,39]. As a matter of facts, models predict that about 20% of the ACh molecules released by the NMJ on repetitive stimulation will diffuse intact out of the cleft [41].

### 2.2. The Case of Nerve-Electroplaque Junction

In certain fish species, a number of muscles are transformed in electric organs. Embryologically, electric organs are therefore modified neuromuscular systems. Despite of a few cytological differences, cholinergic transmission is practically identical at *Torpedo* nerve-electroplaque junctions and at NMJs of various species. Physiological, pharmacological and molecular parameters of transmission are qualitatively and quantitatively close in the two systems. Particularly, the size of evoked ACh quanta and that of the main population of spontaneous quanta is the same (6000–10,000 ACh molecules [42,43]). In both types of junctions, a population of small quanta, (or subquanta, 600–1000 ACh molecules) becomes prominent at high release rates and under specific experimental conditions [30,44]. A *Torpedo* electric organ (weight: 100–300 g) contains myriads of cholinergic synapses (approximately 4–6 × 10^11^ per organ), which operate with an astonishing synchronicity. The duration of a single electric discharge generated by a mean size *Torpedo* is 2–4 ms, delivering in the well-conducting sea water a discharge of approximately 8 A at 15 V. Even at 15 °C, the fish can deliver repetitive discharges at frequencies of 100–200 Hz, for attack or defense. In view of these similitudes between NMJs and NEJs, the abbreviation EPP will be used for both, the end-plate potential and the nerve-electroplaque potential.

**Figure 1:**
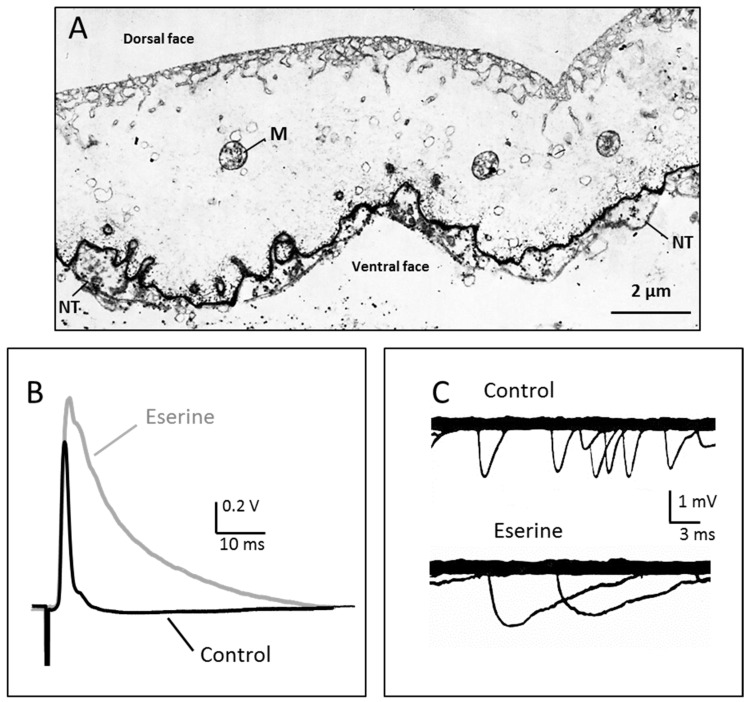
Ultra-rapid transmission and acetylcholinesterase localisation at the nerve-electroplaque junction. (**A**) The *Torpedo* electric organ is a modified nerve-muscle system, where large flat electrocytes (the electroplaques) are covered at their ventral surface by a dense network of nerve terminals (NT). Acetylcholinesterase (AChE, chiefly the asymetric A12 form) is highly concentrated in the synaptic cleft, like in neuro-muscular junctions. M: mitochondriae; (**B**) Transmission of a single impulse is extremely fast in the *Torpedo* nerve-electroplaque junctions (NEJ), as recorded here from a small piece of electric organ in response to a single stimulus applied to afferent nerve fibres. The postsynaptic response (electroplaque potential, or EPP) only lasts 2–3 ms (black trace). AChE inhibition by eserine (10^−4^ M) greatly prolongs the EPP time-course (grey trace); (**C**) Spontaneous miniature potentials recorded at the *Torpedo* NEJ. Their time course is also lengthened when AChE is inhibited. Modified from [47].

Figure 1 shows that like in NMJs acetylcholinesterase is highly concentrated in the synaptic cleft of the nerve-electroplaque junction [12]. In the *Torpedo* electric organ, it is the asymmetric forms (mainly A12), which are present, while the globular G4-AChE is only a minor component [13]. The NEJ can therefore be regarded as a “pure” ultra-fast synapse. As seen in Figure 1, cholinesterase inhibitors markedly prolong the time course of the EPP [45,46], similarly affecting evoked EPPs and spontaneous miniature EPPs [47], just like at the frog NMJ [35]. Unexpectedly, AChE inhibitors markedly reduce the amount of ACh released per impulse. In the presence of eserine, therefore, or after fluostigmine pre-treatment, the release of less ACh generates a larger EPP. The depression of ACh release by cholinesterase inhibitors is mediated by activation of presynaptic muscarinic auto-receptors. This muscarinic auto-inhibition is apparently a general property of cholinergic synapses, occurring in the CNS Szerb [48] as well in the electric organ [49].

### 2.3. Two Presynaptic Mechanisms Which Curtail the Duration of Individual Impulses in Ultra-Fast Cholinergic Synapses

As stated above, the remarkable rapidity of transmission in NMJs and NEJs would be impossible without the strategic localisation of A12-AChE in the synaptic cleft. Two specific “abbreviating” mechanisms are also at work in presynaptic terminals [47]. The first mechanism relies on voltage-gated K^+^-channels which efficiently abbreviate the duration of the presynaptic action potential. At the arrival of a nerve action potential, the axon is abruptly depolarised, due to opening of voltage-operated Na^+^-channels. Then, the nerve is rapidly repolarised (quite before inactivation of the Na^+^-channels) by a fast voltage-operated outward K^+^ current (the so-called delayed rectifier), which rapidly rises to a maximum and then declines slowly [50]. The delayed rectifier is particularly efficient in NMJ and NEJ axon terminals [51,52,53].

The rapid outward K^+^ current can be inhibited by several chemicals, especially the members of the aminopyridine (AmPY) family [54]. At NMJs and NEJs, AmPys dramatically potentiate the duration of ACh release in individual impulses. The EPP is enormously extended, due to prolongation of the inward Ca^2+^-current [55,56]. In the *Torpedo* electric organ, the relatively brief EPP (2–4 ms) is converted into a giant EPP, which abates only after 500–600 ms [46,57]. The shape of individual miniature potentials is not affected by AmPYs, showing that the giant evoked response results from the release of an enormous number of normal quanta [56,58]. AmPYs have practically no effects on the terminals of resting NMJs and NEJ; it is only when the afferents fibres are given a stimulus that the junction generates a giant impulse. The picture is different in CNS nerve terminals, where AmPYs provoke a significant depolarisation in the absence of stimulation and increase spontaneous transmitter release [59]. This results in convulsions and other troubles. Among the members of the AmPY family, the toxicity therefore is related to the ability to cross the blood-brain barrier [60].

The second “abbreviating” mechanism is provided by the vesicular Ca^2+^/H^+^ antiport, which curtails the presynaptic Ca^2+^ spark. Several processes converge towards reducing Ca^2+^ concentration in nerve terminal micro-domains after an action potential. They are diffusion, protein binding, Ca^2+^/Na^+^ exchange, Ca^2+^-pumps in the plasmalemma, the reticulum and synaptic vesicles [61,62,63,64,65]. Most of them are high-affinity processes, therefore poor candidates for a fast repetitive involvement during transmission. However, Gonçalves et al. [66,67] discovered that calcium is also sequestrated into mammalian brain synaptic vesicles via a low-affinity Ca^2+^/H^+^ antiport (K_0.5_ = 217 µM; maximal activity at 500–600 µM Ca^2+^). This vesicular Ca^2+^/H^+^ antiport works in milliseconds. It efficiently curtails the duration of the Ca^2+^ spark during transmission of an individual impulse. As a matter of fact, blocking the vesicular Ca^2+^/H^+^ antiport, a manoeuvre which significantly lengthens the duration of ACh release, results in a EPP of longer time course [68]. The molecular counterpart of the vesicular Ca^2+^/H^+^ antiport was recently elucidated: Synaptotagmin-1 (Syt-1) is essential for its activity. Synaptotagmin-1 is a vesicular protein interacting with membranes upon low-affinity Ca^2+^-binding. It seems to play a major role in excitation-release coupling, by synchronizing calcium entry with fast neurotransmitter release. Experiments carried out with Syt-1-positive and Syt-1 negative cell lines showed that Synaptotagmin-1 is absolutely required for the antiport expression [69]. Further work will be required to decide whether this antiport might be Synaptotagmin-1 itself. 

### 2.4. Other Rapid Protagonists of the Neuro-Muscular Junction and the Nerve-Electroplaque Junction

Most processes involved in rapid neurotransmission are characterised by a typical behaviour: abrupt exposition to the triggering agent (voltage change or ligand binding) causes fast opening followed by fast closure, but in most cases prolonged exposition to the same agent provokes desensitisation. This is well-known for voltage-gated ion channels. They display a brief open time in response to a rapid potential change. Most of them undergo desensitisation if the electrical stimulus is prolonged. Many ligand gated channels exhibit a similar behaviour. For instance, nicotinic ACh receptors (nAChR) in the postsynaptic membrane rapidly open in response to an abrupt jet of ACh (high-speed, low-affinity process). The mean open time of nAChR is about 1 ms at NMJs; it is even shorter (0.6 ms) in the *Torpedo* NEJ [70]. However, if ACh is not rapidly removed, the receptors will desensitize, that is, they will no longer open in response to ACh [71]. 

On the presynaptic side, the behaviour of the mediatophores is quite similar. AThe mediatophore is a proteolipid complex forming clusters at the presynaptic active zones of NEJs, NMJs and other synapses. The mediatophore is are an homo-oligomer composed of several copies of a 15–16 kDa proteolipid which is produced by the *ATP6VOC* gene. The proteolipid is therefore similar to the c-subunit of the membrane sector of the vacuolar ATPase (V-ATPase). When reconstituted in liposomes, oocytes or deficient cell lines, mediatophore mimics the physiological ACh release, including the production of ACh quanta [72,73,74]. The mediatophores release ACh quanta in response to a sudden elevation of [Ca^2+^]_i_, but desensitisation will occur if [Ca^2+^]_i_ remains elevated for several seconds or minutes [75] (see www.mediatophore.ch).

## 3. Cholinergic Transmission in Neuro-Neuronal Synapses

Acetylcholine operates as a neurotransmitter in a majority of neuro-neuronal synapses of the autonomic nervous system, and in a number of synapses of the central nervous systems (CNS). Transmission is cholinergic at these places but there are important differences with NMJ and NEJ transmission: (a) one cholinoceptive neuron usually receives impulses from several afferent nerve fibres; (b) the time course of the initial nicotinic EPSP is 10–50 times slower than the EPP of NMJs and NEJs; (c) the initial EPSP is followed by a complex trail of late responses, some of them being muscarinic, other nicotinic, involving a variety of different mechanisms; (d) there is no A12-AChE concentrated in the synaptic cleft of these synapses. Acetylcholinesterase is present in these structures only as globular forms (mainly G4), which are secreted or membrane bound in the presynaptic, the postsynaptic cells, or in various elements such as glia, vascular endothelium. G4-AChE has not been shown to be concentrated in synaptic clefts.

**Figure 2:**
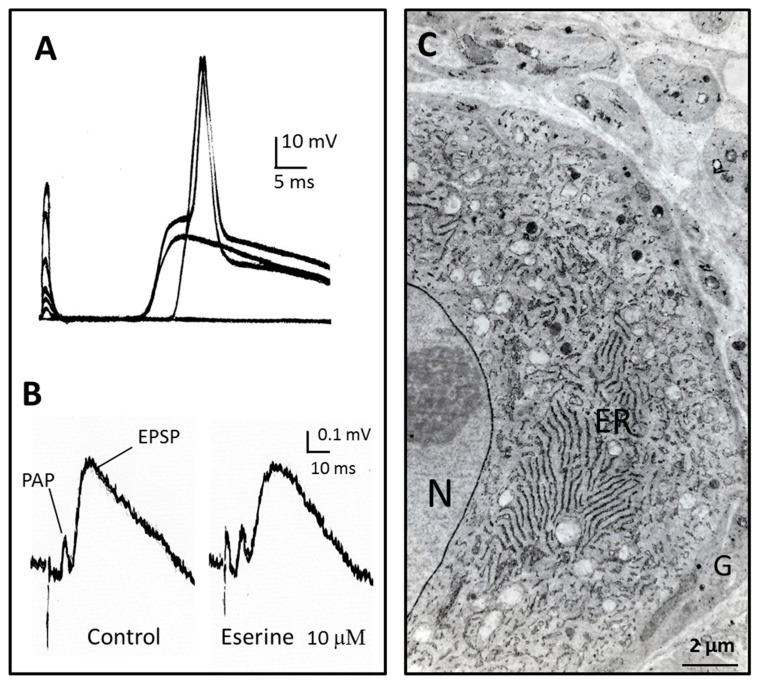
Intermediate time-course of transmission in the rat sympathetic ganglion. (**A**) Excitatory postsynaptic potentials and action potentials recorded intracellularly from a neurone of the rat superior cervical ganglion in response to stimuli of increasing intensity applied to the preganglionic nerve. The different delays after stimulus artefact result from different conduction times in the preganglionic nerve fibres. The initial nicotinic excitatory post synaptic potential (EPSP) lasts for several tens of ms; (**B**) Extracellular recording in a ganglion where transmission was partly blocked by mecamylamine (25 µM). PAP: presynaptic action potential [52]. At the difference of neuro-muscular and nerve-electroplaque junctions, the anticholinesterase eserine does not prolong the EPSP time course; (**C**) AChE localisation in the same preparation. Most of the activity is present in endoplasmic reticulum of neurons (ER) and on the nuclear membrane (N). Glial cells (G) do not show significant AChE histochemical reaction. Most of AChE in ganglia is present as the globular G4-AChE, which is secreted from the neurons. See further explanations and references in the text. Unpublished observations by Y.D., V.G. and Jean Gautron.

Our purpose here is just to delineate some general features of transmission in cholinergic neuro-neuronal synapses. There are of course important differences among different animal species, different ganglia, even among different neurons in the same ganglion. Figure 2 shows intracellular and extracellular traces recorded in a rat superior cervical ganglion in response to stimulation of preganglionic nerve fibres. Obviously, transmission is slower than at the NMJs. The excitatory postsynaptic potential (EPSP) culminates 5–10 ms after the signal marking the excitation of presynaptic nerve terminals (presynaptic action potential, PAP). The EPSP decay then takes several tens of milliseconds. Upon a preganglionic stimulation of graded intensity, the neuron responds either by a lone EPSP, or by EPSPs triggering an action potential. The different delays from the stimulus artefact reveal that several afferent fibres with a distinct conduction time converge to the same neuron (Figure 2A). Extracellular record from a ganglion where nicotinic transmission was partly inhibited by mecamylamine (25 µM) is seen in Figure 2B. Following the stimulus artefact, the compound EPSP is preceded by a brief presynaptic action potential (PAP) [52]. Unexpectedly, the time-course of the initial EPSP was not significantly prolonged when AChE was inhibited by 10 µM eserine. The unavoidable conclusion is that the action of ACh of the initial nicotinic EPSP in ganglionic synapses was not abbreviated by AChE [76]. This is in line with the fact that AChE is not concentrated in the cleft of neuro-neuronal synapses, as it is in the NMJ.

A similar “resistance” of the initial nicotinic EPSP to anticholinesterases was observed in several types of cholinergic neuro-neuronal synapses in the ANS and CNS [77,78,79,80,81,82]. It was proposed that the decay time of the initial EPSP in these systems was compatible with the time taken by ACh to diffuse out of the synapse. Such a discrepancy between eserine action on NMJs and sympathetic ganglia remained nevertheless a problem for a long time. As mentioned above, A12-AChE is practically absent in ANS ganglia and in CNS cholinergic synapses. Instead, AChE is present mainly as the globular form G4, which is predominantly located in the endoplasmic reticulum of neurons (Figure 2C), from where it is secreted in the extracellular space [83].

In denervated ganglia, AChE activity is sharply reduced in parallel with the disruption of the neuron’s endoplasmic reticulum cisternae [84]. These changes following denervation might suggest that AChE is one of the proteins whose synthesis is regulated by activity-induced trans-synaptic mechanisms [85,86]. When the period of denervation is prolonged, a substantial amount of AChE reappears in the ganglion, starting in glial cells. The AChE changes occurring after denervation may exhibit marked differences from one synapse to another and from one species to another [76,84,87,88].

The first example of cholinergic neuro-neuronal synapses in the CNS was described in the spinal cord: the motoneurons/Renshaw-cells synapses [78]. The Renshaw-cells are spinal interneurons which receive a cholinergic nicotinic activation from motoneuron collaterals; they exert a GABA-ergic inhibition on a large pool of motoneurons (GABA, γ-aminobutyric acid). The system therefore realizes a neuronal negative feedback loop. The initial response recorded from Renshaw cells in response to a single volley of motoneuron collaterals lasts a few tens of milliseconds and displays a complex time course. It is first composed of two peaks, generated via two populations of nAChRs. The first peak occurs via fast, low-affinity, α7 nAChRs. The following complex responses are generated by slower, high-affinity, α4β2 nAChRs, and by other mechanisms including currents mediated via glutamate receptors, since glutamate is co-released with ACh [80,89]. In CNS like in ANS ganglia, AChE is mainly present as the tetrameric globular form, G4-AChE, which is membrane-bound and secreted in the vicinity but not densely concentrated in the synaptic cleft. A12-AChE being absent in these synapses, it is not surprising that the time-course of the initial nicotinic EPSP recorded from Renshaw cells is not prolonged when AChE is inhibited or absent. In contrast, addition of AChE abbreviates the late cholinergic responses and anticholinesterases greatly prolongs these late responses, suggesting that they are consecutive to ACh spillover toward extra-synaptic areas. Interestingly, in the absence of G4-AChE these synapses can adapt themselves to the increased local ACh concentration [81]. The adaptation is apparently supported by auto-inhibition of ACh release [48] and by down regulation of receptors.

Whereas the time course of the initial EPSP is not governed by ACh hydrolysis in most cholinergic synapses, G4-AChE is in a position to hydrolyse the fraction of ACh which spills over from synapses. Doing this, AChE—which is provided locally in a secreted form—will regulate the late effects of ACh on extra-synaptic receptors. It will also furnish choline for local ACh re-synthesis [87].

## 4. The Great Diversity of Slow Cholinergic Processes in the Central and Autonomic Nervous Systems

### 4.1. Postganglionic Parasympathetic Nervous System

The first demonstrations of ACh being a mediator substance were performed using organs that are target of parasympathetic postganglionic fibres, such as cardiac and smooth muscle cells, secretory cells, etc. [1,2,3]. It has long been known that transmitter action in these tissues is not a brief and highly restricted point-to-point process, and that ACh acts mostly via “metabotropic” muscarinic receptors (mAChRs), which have a relatively high affinity for ACh and are coupled to G-proteins. Innervation of the target organs is loose, with bunches of terminals and varicosities spraying ACh over a relatively vast territory. The time between ACh release and the end of the cellular effects in these areas is by several orders of magnitude longer than at the NMJ, ranging from seconds to minutes, or more. In others words, the cholinergic signalling is not phasic, but tonic. ACh at these places should no longer be regarded as a neurotransmitter but as a local hormone [90]. We don’t want to elaborate on this field, but just to recall it since cholinergic mechanisms in the CNS resemble on many aspects those of postganglionic parasympathetic nervous system.

### 4.2. Central Nervous System

The identification and understanding of cholinergic processes in the CNS remained limited until recently due to the complexity and diversity of mechanisms involved. However, marked progresses have resulted from new experimental approaches, such as immune-localisation of choline acetyltransferase (ChAT), AChE and other synaptic proteins, improved ACh assays combined to push-pull cannula and electrophysiology, molecular biology, localisation of muscarinic and nicotinic receptors, optogenetic analysis of synaptic transmission, etc. 

#### 4.2.1. Relative Paucity of “True” Cholinergic Synapses in the Central Nervous System

As mentioned above, typical cholinergic rapid synapses resembling those of the ANS ganglia exist in the CNS: we mentioned the motoneurons/Renshaw cells synapses of the spinal cord [78,89]. Recently, refined investigations revealed that cholinergic synapses are more abundant in the CNS that was anticipated. Small cholinergic synapses in which a thin post-synaptic density contains neuroligin 2 were identified in the cortex. Transmission in these synapses was analysed using optogenetic approaches and found to work in a complex manner as described above [79,82,91,92,93]. However, a large number of cholinergic terminals, boutons and varicosities which were systematically examined at the ultrastructural level, lacked the junctional membrane specialisations (close apposition of pre- and postsynaptic membranes, local membrane thickenings), that are the hallmark of synapses (see review in [16]).

#### 4.2.2. Many Nicotinic and Muscarinic Acetylcholine Receptors in the Brain Are Extrasynaptic

Cytological examination of the immediate microenvironment of cholinergic terminals revealed that the majority of ACh receptors, either muscarinic (mAChRs) or nicotinic (nAChRs) are not precisely localised on the postsynaptic thickenings, as could be expected. They are mainly present in extra-junctional areas of neurons, dendrites, axons and axon endings (of cholinergic and non-cholinergic cells), as well as on astroglia and microvessels (see reviews in [94,95,96]).

In most instances, the messages conveyed by ACh in the brain are slow. For instance, cortical neurons respond to a iontopheretic application of ACh by a delayed excitation which can last several seconds [97]. Presynaptic mAChR at several places inhibits the release of various transmitters, including ACh [48], while activation of presynaptic or pre-terminal nAChRs was found to elicit the release of transmitters. As for the nicotinic receptors, most homomer α7 nAChRs have a relatively low affinity for ACh whereas heteromers (mainly α4β2) nAChRs have a much higher affinity. Both types are subject to desensitisation upon sustained exposure to agonists [94,95,96]. For instance, nicotine application to hippocampus mossy fibres causes a delayed and prolonged release of glutamate. The process occurs without membrane depolarisation and without dissipation of the vesicular proton gradient [98,99].

#### 4.2.3. Acetylcholinesterase Forms in the Central Nervous System

The major forms of AChE in CNS of birds and mammals are the globular forms, mainly G4-AChE. In contrast, collagen-tailed forms such as A12-AChE are barely detectable in the brain of higher vertebrates. However, A12-AChE was reported to be abundant in the brain of lower vertebrates like fish or frogs [100]. G4-AChE in the mammalian brain is predominantly amphiphilic and membrane-bound, only 10–20% being non-amphiphilic. AChE is relatively abundant on the membrane of cholinergic neurons (identified by ChAT activity) but it is also found in other types of neurons and on non-neuronal cells (glia). In certain areas, there is a complete mismatch between AChE and ChAT localisation. G4-AChE has been shown to be secreted in several areas of the CNS and the rate of secretion is modulated by neuronal activity, changes in neurotransmitter levels or drug treatments. As a consequence, significant amounts of AChE can be measured in the cerebrospinal fluid [16,101].

#### 4.2.4. Diffuse or “Volume” Transmission Seems to Be the Major Mode of Acetylcholine Action in the Central Nervous System

The data obtained by direct estimation of the ACh concentration in different brain areas support the concept that ACh acts more as a local hormone in the CNS than as a rapid point to point neurotransmitter. The mediator is released from bunches of cholinergic boutons and varicosities, it diffuses and exerts its effects on several cells in a relatively large volume. The local ACh concentration is a balance between the rate of release and the rate of hydrolysis by the membrane-bound or secreted G4-AChE. At the exception of the limited number of synapses where ACh transmits nerve impulses at a relatively high rapidity (a phasic process), cholinergic transmission in the CNS appears mainly as a tonic process, where a steady concentration of ACh is controlled in space and time by an extra-synaptic cholinesterase [16,101].

## 5. Conclusions

We have reviewed ACh mediation in vertebrates with particular attention to the time-course of processes. The picture emerging by confronting recent and old data is just at the opposite of the classical description of the cholinergic kingdom. Textbooks claim that ACh is a fast neurotransmitter; they present the neuromuscular junction as the model of cholinergic synapses, but the vertebrate neuromuscular junction is an exception, which is specialised for high-speed transmission. In the vast majority of cells and organisms, including most sectors of the human CNS, acetylcholine is a local hormone; its secretion can be qualified of paracrine or autocrine, generating relatively slow effects. Among the factors differencing fast and slow cholinergic transmission, the principal is the cytological organisation: close apposition of pre- and post-synaptic membranes vs. a loose regional innervation of a vast group of cells. Acetylcholinesterase and its molecular forms are differently involved in slow, rapid and ultra-fast cholinergic signalling. Thanks to its strategical localisation, A12-AChE hydrolyses ACh at a very high speed, abbreviating individual impulses at NMJs and NEJs. This is also an exception. In most places, G4-AChE regulates ACh concentration and achieves hydrolysis over larger areas, with a slower time course.

Less is known concerning potential differences between the presynaptic mechanisms engaged in fast and slow cholinergic mediation. We showed that certain presynaptic K^+^ channels (delayed rectifier) and the vesicular Ca^2+^/H^+^ antiport are crucial for making very brief the ACh release in individual impulses. Are they also at work in slower cholinergic signalling? Mediatophores and other products of the *ATP6VOC* gene are implicated in several cellular mechanisms, including quantal transmitter release and membrane fusion [73,102]. Mediatophorea are also responsible for ACh release in non-neuronal systems, such as T lymphocytes [6]. Being also involved in the release of other transmitters, mediatophores have been recently proposed as a promising tool for gene therapies of neuro-degenerative diseases [103]. Acetylcholine, though a very simple molecule, bears the ability to control a wide spectrum of functions, ranging from ultra-fast transmission to paracrine diffuse impact. Due to its ubiquity and complexity, the “old” field of acetylcholine is still open to fascinating explorations.

## Abbreviations

ACh	acetylcholine
AChE	acetylcholinesterase
mAChRs and nAChRs	muscarinic and nicotinic ACh receptors, respectively
AmPy	aminopyridine
ANS and CNS	autonomic and central nervous system, respectively
ChAT	choline acetyltransferase
EPP	endplate potential or electroplaque potential
EPSP	excitatory postsynaptic potential
MEPPs	miniature endplate or miniature electroplaque potentials
NEJ	nerve-electroplaque junctions of electric organs
NMJ	neuromuscular junction
PRiMA	proline-rich membrane anchor
